# Immediate gratification and continued medical insurance preparedness: the roles of future orientation and relative deprivation

**DOI:** 10.3389/fpubh.2026.1987239

**Published:** 2026-10-19

**Authors:** Long Shi, Qizhe Zhao, Hongcheng Liu, Wenxu Li, Panzhen Zhao, Caisheng Liao

**Affiliations:** 1 School of Humanities and Management, Youjiang Medical University for Nationalities, Baise, China; 2 School of Finance, City University of Macau, Macau, China; 3 Graduate School of Medical Sciences, Kanazawa University, Kanazawa, Ishikawa, Japan; 4 School of Medical and Nursing, Shandong Vocational University of Foreign Affairs, Weihai, China; 5 School of Languages and Cultures, Youjiang Medical University for Nationalities, Baise, China; 6 Division of Electrical, Information and Communication Engineering, Kanazawa University, Kanazawa, Japan

**Keywords:** future orientation, immediate gratification, medical insurance, relative deprivation, socioemotional selectivity theory

## Abstract

**Background:**

Sustained participation in medical insurance is essential for the effectiveness and stability of social protection systems. Despite high coverage rates, many individuals show unstable enrollment patterns, characterized by repeated interruptions and re-entry. Existing research has mainly focused on institutional and economic explanations, while the psychological mechanisms underlying continued insurance preparedness remain underexplored. Drawing on socioemotional selectivity theory, this study examines how immediate gratification relates to continued insurance preparedness through future orientation, with relative deprivation as a key boundary condition.

**Methods:**

A dual-study research design was adopted. Study 1 employed a scenario-based experiment with a 2 × 2 between-subjects design to vary immediate-gratification and relative-deprivation framing and examine differences in future-orientation responses across conditions using two-way analysis of covariance (ANCOVA), controlling for gender, age, living allowance, and hukou. Study 2 utilized survey data collected from urban and rural residents in Guangxi, China, and applied PLS-SEM to test a moderated mediation model linking immediate gratification, future orientation, relative deprivation, and continued insurance preparedness.

**Results:**

Study 1 showed that high immediate-gratification framing was associated with lower future orientation than low immediate-gratification framing, with this difference becoming larger under high relative deprivation. Study 2 further showed that immediate gratification was negatively associated with future orientation, which in turn was positively associated with self-reported continued insurance preparedness. Although the direct association between immediate gratification and preparedness was not significant, the indirect association through future orientation was significant and became stronger as relative deprivation increased. Multigroup analysis further suggested a stronger indirect association among rural-hukou respondents than among urban-hukou respondents, although the between-group difference did not remain statistically significant after adjustment for multiple comparisons.

**Conclusion:**

Continued insurance preparedness was closely associated with future-oriented cognition, while immediate gratification was indirectly associated with lower preparedness through lower future orientation, particularly under higher relative deprivation. These findings advance understanding of the psychological correlates of self-reported readiness to maintain medical insurance participation and provide practical insights for supporting future-oriented enrollment planning.

## 1 Introduction

Medical insurance participation among urban and rural residents constitutes a critical precondition for the social security system to fulfill its functions of risk sharing and redistribution. In recent years, with the continuous improvement of China’s basic medical insurance system, the participation coverage rate has been maintained at a consistently high level; however, challenges remain regarding the stability of continuous contributions and long-term enrollment behavior (1, 2). In particular, amid increasingly flexible employment arrangements and the pervasive influence of consumerism, certain groups exhibit a stronger preference for immediate consumption and short-term gratification, thereby placing long-term, security-oriented behaviors in a secondary position (3, 4). At the same time, the sustained rise in household leverage ratios and the rapid expansion of consumer credit in China further reflect the growing prevalence of present-oriented consumption patterns (5, 6). Against this backdrop, many urban and rural residents continue to exhibit recurrent cycles of insurance interruption, re-enrollment, and subsequent interruption, a pattern that is particularly pronounced among migrant populations, flexibly employed workers, and younger cohorts (7, 8). This repeatedly observed behavioral pattern suggests that sustained medical insurance participation is shaped not only by institutional arrangements and economic conditions, but also by psychological processes underlying intertemporal decision-making.

Existing research has extensively examined insurance participation from structural and economic perspectives. From a structural perspective, the household registration system, occupational stratification, disparities in institutional accessibility, and mechanisms of social exclusion have been shown to significantly affect both enrollment opportunities and continuity (9–11). From an economic perspective, income level, employment stability, and contribution burden are widely regarded as key constraints shaping participation decisions, rendering enrollment behavior particularly sensitive to income fluctuations and external economic pressures (12, 13). More recently, research has extended beyond these structural and economic determinants to consider individual-level psychological and decision-making factors, including risk aversion, institutional trust, financial literacy, and subjective expectations (14–16). Intertemporal preferences have also received increasing attention. For example, Baillon et al. (17) showed that time preferences were associated with health insurance uptake, while highlighting the importance of liquidity constraints in shaping this relationship. However, existing research has focused primarily on explaining enrollment, uptake, or enrollment intentions, leaving the cognitive process through which present-focused preferences are associated with individuals’ readiness to maintain insurance participation over time. In particular, the role of future-oriented cognition in linking short-term preferences to continued insurance preparedness remains insufficiently examined.

To address this gap, this study examines how immediate gratification relates to future orientation and how this process carries consequences for continued insurance preparedness. Socioemotional selectivity theory (SST) suggests that perceptions of future time shape goal priorities, with more expansive time horizons favoring preparatory and future-oriented goals and more limited horizons increasing the priority of goals realized in the present (18, 19). Consistent with this temporal trade-off, future-oriented thinking has been associated with greater willingness to forgo immediate gratification in favor of delayed rewards (20). Importantly, this relationship may become stronger under relative deprivation, which has been linked to greater materialistic priorities and stronger preferences for smaller-sooner rewards (21–23). Unlike objective liquidity constraints or peer effects arising from others’ observed financial choices (24), relative deprivation reflects a subjective perception of disadvantage arising from social comparison. In turn, weaker future orientation may undermine continued insurance preparedness because sustained participation requires individuals to weigh current contributions against future protection, consistent with evidence linking future time perspective to insurance purchasing (25). Accordingly, we propose that relative deprivation strengthens the negative relationship between immediate gratification and future orientation, rather than merely reflecting limited economic resources or direct social influence, with downstream consequences for continued insurance preparedness.

Based on this framework, the study uses two complementary designs. Study 1 employs a scenario-based experiment to examine differences in future-orientation responses across immediate-gratification conditions and whether these differences vary by relative-deprivation condition. Study 2 uses survey data from urban and rural residents to test whether this conditional psychological process extends to continued insurance preparedness. Together, the two studies complement existing structural and economic explanations of insurance participation by examining the role of intertemporal psychological factors in continued insurance preparedness. Specifically, they focus on future orientation as a potential pathway linking immediate gratification to preparedness and on relative deprivation as a condition under which this association may become stronger.

## 2 Literature review and hypotheses

### 2.1 Immediate gratification and future orientation

Immediate gratification reflects a present-focused tendency to prioritize immediate concerns and outcomes over delayed consequences (26, 27), whereas future orientation refers to the extent to which individuals incorporate future consequences into current decision-making (28). Although negatively related, the two constructs are not opposite ends of a single continuum: immediate gratification concerns the valuation of immediate rewards, whereas future orientation concerns attention to future consequences. Thus, individuals may recognize future consequences while still preferring immediate rewards.

SST provides a useful temporal perspective for understanding this contrast. The theory proposes that perceived time horizons shape goal priorities: relatively open-ended time horizons increase the priority of knowledge-oriented and preparatory goals, whereas constrained time horizons increase the priority of emotionally meaningful goals in the present (18, 19). Consistent with the broader distinction between present and future-focused orientations, empirical research also shows that present-hedonistic time perspectives are associated with a lower willingness to delay gratification (20). Applied to the present context, temporal focus may shape the relative priority assigned to goals whose benefits are realized in the present versus those that require preparation for future outcomes. Accordingly, greater emphasis on immediate outcomes is expected to be associated with less consideration of future consequences and lower future orientation. Accordingly, this study proposes:

*H1*: Immediate gratification is negatively associated with future orientation.

### 2.2 Relative deprivation as a boundary condition

Relative deprivation refers to the perception that one is worse off than a relevant comparison standard, accompanied by feelings of resentment and unfairness (21, 29). Importantly, relative deprivation can influence how individuals evaluate outcomes across time. In a series of studies, Callan et al. (21) found that experimentally induced personal relative deprivation increased preferences for smaller-sooner over larger-later rewards, indicating a greater tendency to discount delayed outcomes. Related evidence indicates that personal relative deprivation is associated with future discounting, higher impulsivity, and lower self-control (30). More recently, Pan and Zhao (23) found that relative deprivation formed part of the psychological process through which upward social comparison was associated with a greater preference for smaller, more immediate rewards over larger, delayed rewards. These findings suggest that relative deprivation can shift intertemporal valuation toward outcomes available in the present and away from outcomes whose benefits lie further in the future.

Relative deprivation can therefore be understood as a contextual condition that alters the temporal weighting of competing goals. From the perspective of SST, goal priorities depend on the relative salience of present versus future outcomes (18, 19). When relative deprivation is high, the stronger preference for smaller-sooner rewards and greater discounting of delayed outcomes associated with deprivation make present-oriented goals more salient (21–23). Under these conditions, the present-focused tendency inherent in immediate gratification is more likely to dominate decision-making, reducing the psychological weight assigned to future consequences and long-term goals. By contrast, when relative deprivation is low, this additional pressure toward immediate outcomes is weaker, allowing future-oriented considerations to retain greater salience. Accordingly, we propose:

*H2*: Relative deprivation moderates the negative relationship between immediate gratification and future orientation, such that the relationship is stronger when relative deprivation is high rather than low.

### 2.3 Conditional indirect association with continued insurance preparedness

Continued insurance preparedness reflects individuals’ readiness to maintain or resume insurance participation and to plan for future insurance-related expenditures. Under China’s urban and rural residents basic medical insurance, coverage is financed jointly through individual contributions and government subsidies, while insurance benefits are provided primarily through reimbursement for eligible medical expenditures (31, 32). The relevant intertemporal trade-off therefore does not involve exchanging a present cost for a certain delayed reward. Rather, a current contribution helps maintain financial protection against uncertain future medical expenditure, with insurance benefits becoming relevant when covered healthcare needs arise (33, 34). Future orientation is particularly relevant because it enables individuals to incorporate future medical needs and the consequences of maintaining coverage into current planning. Prior research has linked stronger future time perspectives to greater financial preparedness for retirement (28) and a higher propensity to purchase several forms of insurance (25). Thus, when future-oriented cognition is weakened, individuals may attach less weight to future coverage needs, reducing their preparedness to maintain participation across subsequent enrollment periods.

The downstream consequences of this weakened future orientation should become particularly consequential when immediate rather than delayed outcomes dominate decision-making. Evidence from financial behavior shows that present-biased individuals tend to save less and borrow more, indicating that greater emphasis on current rewards can undermine behaviors requiring longer-term commitment (3). At the same time, relative deprivation has been shown to reduce engagement in future-beneficial behaviors and to lower the relative priority assigned to longer-term health goals (35). These findings suggest that when relative deprivation is high, the reduction in future orientation associated with immediate gratification is more likely to carry forward into decisions requiring sustained commitment. Consequently, immediate gratification should be associated with lower continued insurance preparedness through future orientation, with this negative indirect relationship becoming stronger as relative deprivation increases. Accordingly, we propose:

*H3*: Relative deprivation moderates the indirect relationship between immediate gratification and continued insurance preparedness through future orientation, such that the negative indirect relationship is stronger when relative deprivation is high rather than low.

Based on the above hypotheses, Figure 1 presents the conceptual model of this study, illustrating how immediate gratification is linked to continued insurance preparedness through future orientation and how this indirect process varies with relative deprivation.

**Figure 1:**
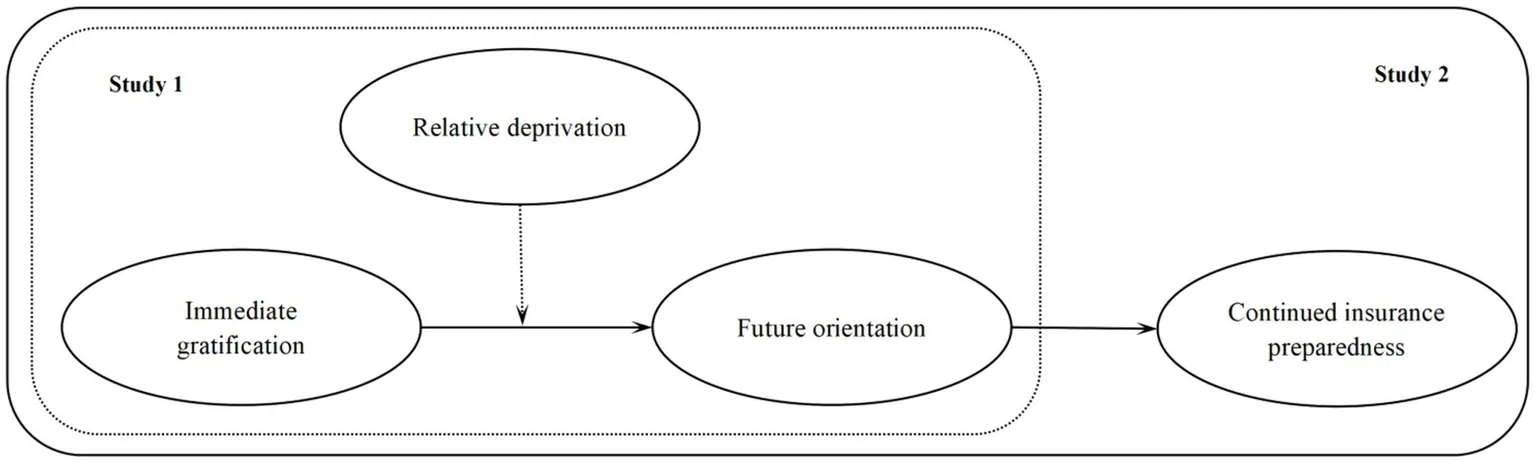
Conceptual model of this study.

## 3 Methodology

### 3.1 Research design

To examine the proposed framework, this study adopts two complementary empirical designs. Study 1 uses a 2 × 2 scenario-based experiment varying immediate-gratification and relative-deprivation framing and provides preliminary evidence regarding differences in future-orientation responses across these conditions, corresponding to H1 and H2. Study 2 uses survey data from urban and rural residents and applies partial least squares structural equation modeling (PLS-SEM) to examine the full model at the perceptual level, including the conditional indirect association with continued insurance preparedness. The two studies examine the proposed psychological process using complementary experimental and survey-based approaches. An overview of the two-study design is provided in Table 1.

**Table 1:** Overview of the two-study design.

Study	Design and sample	Primary analysis	Main purpose
Study 1	2 × 2 scenario experiment; university students (*N* = 211)	Two-way analysis of covariance (ANCOVA)	Examine future orientation responses across immediate-gratification and relative-deprivation framing conditions
Study 2	Survey; urban and rural residents (*N* = 465)	PLS-SEM	Examine the full model at the perceptual level, including the conditional indirect relationship with continued insurance preparedness

### 3.2 Measures

The focal constructs were operationalized differently across the two studies in accordance with their respective research designs. In Study 1, immediate gratification and relative deprivation were operationalized through experimentally assigned scenario conditions, whereas future orientation was assessed as the primary outcome. After completing the future-orientation measure, participants responded to scenario-referenced items assessing immediate gratification and relative deprivation. These items served solely as manipulation checks to determine whether participants responded to the experimental conditions in the intended direction. The manipulation-check scores were not used as continuous predictors or moderators in the primary analyses; instead, participants’ randomly assigned experimental conditions were used in the two-way analysis of covariance. In Study 2, by contrast, immediate gratification, future orientation, relative deprivation, and continued insurance preparedness were all measured as continuous individual-level constructs and incorporated into the structural model.

The scale items were adapted from established measures and contextualized to the medical insurance setting. Immediate gratification was adapted from Joireman et al. (26) and captures a preference for immediate benefits and present needs over delayed outcomes. This measure reflects present-oriented reward preferences rather than temporal preference reversals or time inconsistency. Future orientation was adapted from Hershey and Mowen (28) and assesses the extent to which individuals consider future medical needs and longer-term consequences in current decision-making. Relative deprivation was adapted from the Personal Relative Deprivation Scale developed by Callan et al. (21) and reflects perceived disadvantage, unfairness, and dissatisfaction arising from comparisons between one’s own medical insurance protection and that available to comparable others. Continued insurance preparedness, which was assessed only in Study 2, was developed by integrating and contextualizing measures from Mamun et al. (36) and Hershey and Mowen (28). It captures respondents’ readiness to maintain medical insurance coverage over time, including continuation intentions and financial preparation for future insurance-related expenditures. Conceptually, future orientation reflects how future consequences are incorporated into current judgment, whereas continued insurance preparedness reflects domain-specific readiness for continued participation.

Although the manipulation-check items used in Study 1 were adapted from the same underlying construct domains assessed in Study 2, their methodological roles differed across the two studies. In Study 1, the immediate-gratification and relative-deprivation items were explicitly framed with reference to the assigned scenario and were used only to assess manipulation effectiveness. In Study 2, the corresponding items were framed in terms of respondents’ usual tendencies and naturally occurring perceptions and were therefore used as continuous measures of the focal constructs. Future orientation was likewise assessed in a manner appropriate to each study context: the Study 1 items referred to participants’ judgments within the experimental scenario, whereas the Study 2 items reflected respondents’ general medical insurance decision-making.

All scale-based responses were recorded on 7-point Likert scales, with reverse-coded items recoded before analysis so that higher scores consistently indicated higher levels of the corresponding constructs. The complete English and Chinese item wording, response anchors, and scoring procedures are provided in Supplementary material. To ensure contextual appropriateness and semantic equivalence, the questionnaire underwent translation and back-translation, semantic equivalence checks, and pretesting.

## 4 Study 1

### 4.1 Participants and procedure

Study 1 employed an offline scenario-based experimental design and recruited university students as participants. The experiment was conducted in September 2025 at an undergraduate institution in Guangxi, China. Participants were recruited from this institution through course announcements and in-class invitations. Participants received a notebook as compensation for their participation. All experimental sessions were conducted in a standardized classroom environment to ensure consistency in experimental procedures and information presentation. A total of 240 students were recruited, with 60 participants randomly assigned to each of the four experimental conditions. Before the formal experiment, participants were provided with the materials corresponding to their assigned condition and given a brief introduction to the procedure. Participants then proceeded to the experimental tasks following the provided instructions. Based on pre-established data quality criteria, responses that failed the attention checks, were incomplete, or clearly did not comply with the experimental requirements were excluded. Specifically, 8 participants were excluded from the low relative deprivation × low immediate gratification condition, 8 from the low relative deprivation × high immediate gratification condition, 6 from the high relative deprivation × low immediate gratification condition, and 7 from the high relative deprivation × high immediate gratification condition. The final sample consisted of 211 responses included in the subsequent analyses (mean age = 20.284 years; 58.8% female), with final cell sizes of 52, 52, 54, and 53, respectively.

The experiment adopted a 2 (immediate-gratification framing: high vs. low) × 2 (relative-deprivation framing: high vs. low) between-subjects design. Participants were randomly assigned to one of four experimental conditions and independently read one integrated medical insurance decision scenario corresponding to their assigned condition. The immediate-gratification framing varied the temporal emphasis of the scenario: the high immediate gratification condition emphasized immediate enjoyment and short-term benefits, whereas the low immediate gratification condition emphasized future medical risks, long-term protection, and preparation. The relative-deprivation framing varied comparative information about medical insurance benefits: the high deprivation condition described less favorable insurance benefits relative to comparable peers, whereas the low deprivation condition depicted broadly comparable levels of coverage. The four scenario versions followed the same structure and presentation, with wording varied to instantiate the two framing factors. Each participant was presented with only one integrated scenario, and no information regarding condition labels, construct names, or study hypotheses was disclosed. After reading the assigned scenario, participants first completed the future orientation measure. They then completed the immediate gratification and relative deprivation measures, which served as manipulation checks, followed by an attention check and demographic questions.

### 4.2 Manipulation check

Independent-samples *t*-tests were conducted to assess the effectiveness of the experimental manipulations. Participants in the high relative deprivation condition reported significantly higher perceived relative deprivation (*M* = 5.641, SD = 0.668) than those in the low relative deprivation condition (*M* = 2.833, SD = 0.606), *t*(209) = 31.961, *p* < 0.001. Similarly, participants in the high immediate gratification condition reported significantly higher perceived immediate gratification (*M* = 4.880, SD = 0.723) than those in the low immediate gratification condition (*M* = 2.652, SD = 0.678), *t*(209) = 23.088, *p* < 0.001. These results confirmed that participants perceived the two scenario framings in the intended directions. However, because the manipulation checks assessed the intended scenario-referenced constructs, they do not rule out other effects of the scenarios, such as differences in future-oriented information salience or perceived insurance value.

### 4.3 Hypothesis testing

After controlling for gender, age, living allowance, and hukou, a two-way analysis of covariance (ANCOVA) was conducted with future orientation as the dependent variable. The results revealed a significant main effect of the immediate gratification condition on future orientation, *F*(1, 203) = 34.895, *p* < 0.001, partial η^2^ = 0.147. Before covariate adjustment, future orientation was higher in the low immediate gratification condition (*M* = 5.050, SD = 1.376) than in the high immediate gratification condition (*M* = 3.650, SD = 1.183). The corresponding adjusted marginal means were 5.061 [SE = 0.147, 95% CI (4.771, 5.352)] and 3.627 [SE = 0.148, 95% CI (3.336, 3.918)], respectively. Thus, H1 was supported.

The interaction between the immediate gratification and relative deprivation conditions was also significant, *F*(1, 203) = 14.344, *p* < 0.001, partial *η^2^* = 0.066. As shown in Figure 2, under low relative deprivation, the adjusted mean of future orientation was 4.448 [95% CI (4.063, 4.832)] in the low immediate gratification condition and 3.694 [95% CI (3.359, 4.030)] in the high immediate gratification condition. Under high relative deprivation, the corresponding adjusted means were 5.675 [95% CI (5.309, 6.042)] and 3.560 [95% CI (3.103, 4.016)]. Simple-effect comparisons showed that the difference in future orientation between the low and high-immediate-gratification conditions was significant under low relative deprivation [ΔM = 0.754, 95% CI (0.234, 1.273), *p* = 0.005] and became substantially larger under high relative deprivation [ΔM = 2.116, 95% CI (1.452, 2.779), *p* < 0.001]. Thus, the negative relationship between immediate gratification and future orientation was stronger when relative deprivation was high, supporting H2.

**Figure 2:**
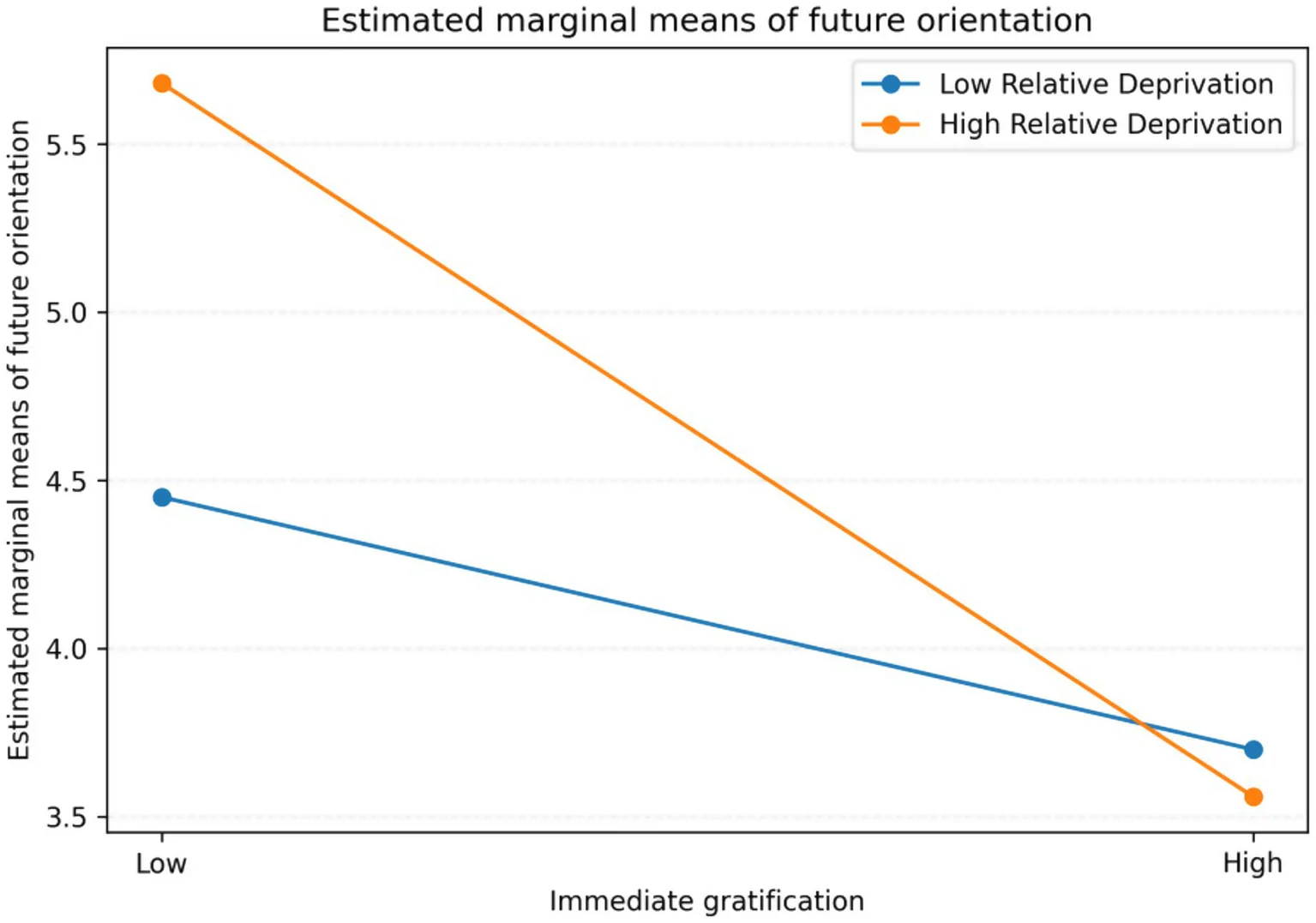
Interaction between immediate gratification and relative deprivation conditions on future orientation. Points represent covariate-adjusted estimated marginal means.

## 5 Study2

### 5.1 Participants and procedure

Study 2 employed a questionnaire-based survey conducted in October 2025 in Guangxi, China. Guangxi was selected as the study setting because its pronounced urban–rural disparities and uneven socioeconomic and healthcare conditions provide a relevant context for examining continued insurance preparedness across urban and rural residents. The survey focused specifically on the basic medical insurance for urban and rural residents, which is organized around recurring annual enrollment, contribution, and coverage periods. Residents generally make an individual contribution during the concentrated enrollment period for coverage in the following year, with government subsidies also contributing to the financing of the scheme. This recurring enrollment arrangement makes decisions about whether to maintain insurance participation particularly relevant to the present study.

To ensure that respondents had sufficient experience with the scheme to evaluate questions concerning coverage and future participation, eligibility required them to be aged 18 years or older, currently reside in Guangxi, and report that they were currently enrolled or had previously been enrolled in the scheme. A non-probability, multi-channel sampling strategy was adopted, and the online questionnaire was distributed through community-based WeChat groups, urban and rural resident discussion groups, and the social networks of university students and their families.

Before completing the survey, participants provided informed consent and were informed that participation was voluntary, responses were anonymous, and the data would be used solely for academic purposes. A total of 526 questionnaires were collected. Respondents who did not meet the eligibility criteria, failed the attention checks, completed the questionnaire in an excessively short time, or provided clearly invalid responses were excluded, leaving 465 valid questionnaires for analysis. The final sample had a mean age of 31.230 years (SD = 8.422); 51.6% were female and 48.4% were male, while 41.3% had urban hukou and 58.7% had rural hukou.

### 5.2 Common method bias(CMB) assessment

To assess common method bias (CMB), two diagnostic procedures were employed. First, Harman’s single-factor test showed that the first unrotated factor accounted for 33.0% of the total variance, below the commonly used 50% threshold (37). Second, the common method factor analysis showed that the average variance explained by the substantive constructs (0.648) was substantially greater than that explained by the method factor (0.005), while the method factor loadings were generally small and of limited statistical significance (38). These results suggest that CMB is unlikely to substantially affect the findings. Nevertheless, because both procedures have inherent limitations, the possibility of some common method influence cannot be completely ruled out.

### 5.3 Evaluating the measurement model

The reliability and validity of the measurement model were systematically assessed in this study. As shown in Table 2, internal consistency was first examined, and the results indicated that Cronbach’s *α* values for all latent constructs exceeded 0.80, while composite reliability (CR) values were above 0.70, demonstrating satisfactory internal consistency. With respect to convergent validity, the average variance extracted (AVE) values for all constructs were greater than the recommended threshold of 0.50 (39). Although a few measurement indicators exhibited outer factor loadings slightly below 0.70, all loadings were above 0.60, and both CR and AVE values met the recommended criteria, indicating acceptable convergent validity. Regarding discriminant validity, as reported in Table 3, inter-construct correlations were lower than the square roots of the corresponding AVE values, and all HTMT ratios were below 0.85, suggesting adequate discriminant validity among the constructs (40).

**Table 2:** Assessment of convergent validity and internal consistency.

Construct	Item	Outer loading	Cronbach’s α	CR	AVE
Continued insurance preparedness (CIP)	CIP1	0.849	0.862	0.906	0.707
	CIP2	0.833			
	CIP3	0.848			
	CIP4	0.833			
Future orientation (FO)	FO1	0.873	0.870	0.911	0.720
	FO2	0.849			
	FO3	0.865			
	FO4	0.807			
Immediate gratification (IG)	IG1	0.842	0.875	0.903	0.574
	IG2	0.788			
	IG3	0.803			
	IG4	0.786			
	IG5	0.668			
	IG6	0.777			
	IG7	0.611			
Relative deprivation (RD)	RD1	0.813	0.863	0.900	0.644
	RD2	0.776			
	RD3	0.754			
	RD4	0.839			
	RD5	0.829			

**Table 3:** Discriminant validity of the measurement model.

Constructs	Mean	S. D.	Fornell-larcker criterion				HTMT ratio			
			1	2	3	4	1	2	3	4
1. CIP	4.327	1.395	**0.841**				/			
2. FO	4.448	1.407	0.449	**0.849**			0.517			
3. IG	3.786	1.096	−0.271	−0.504	**0.757**		0.305	0.557		
4. RD	4.320	1.290	0.219	0.260	−0.301	**0.803**	0.248	0.293	0.335	/

The bold elements are the square roots of AVE; CIP Continued insurance preparedness; FO Future orientation; IG Immediate gratification; RD Relative deprivation.

### 5.4 Hypothesis testing

Before hypothesis testing, the quality of the structural model was assessed in terms of collinearity, explanatory power, and predictive relevance. The VIF values for the structural model ranged from 1.004 to 1.355, which were well below the recommended threshold of 5.0, indicating that multicollinearity was not a concern (39). The *R*^2^ values were 0.207 for continued insurance preparedness and 0.278 for future orientation, indicating acceptable explanatory power. In addition, the *Q^2^*predict values were 0.066 for continued insurance preparedness and 0.264 for future orientation. Both values were greater than 0, supporting the predictive relevance of the model.

Gender, age, educational level, and average monthly income were included as control variables for continued insurance preparedness. The structural model results are presented in Table 4. Immediate gratification was significantly and negatively associated with future orientation (*β* = −0.443, *p* < 0.001), indicating that individuals with stronger immediate-gratification tendencies exhibited lower levels of future orientation. Therefore, H1 was supported. Future orientation was, in turn, significantly and positively associated with continued insurance preparedness (*β* = 0.418, *p* < 0.001). Although the direct association between immediate gratification and continued insurance preparedness was not statistically significant (*β* = −0.062, *p* = 0.235), its indirect association through future orientation was significant (*β* = −0.185, *p* < 0.001). These results support the hypothesized indirect association through future orientation.

**Table 4:** Structural model and hypothesis testing results.

Path/effect	*β*	LLCI	ULCI	*p*-value	VIF	Significance
Direct and indirect effects						
IG → CIP	−0.062	−0.164	0.038	0.235	1.355	No
IG → FO	−0.443	−0.516	−0.369	< 0.001	1.159	Yes
FO → CIP	0.418	0.320	0.513	< 0.001	1.344	Yes
IG → FO → CIP	−0.185	−0.244	−0.134	< 0.001	—	Yes
Moderating and moderated indirect effects						
RD × IG → FO	−0.113	−0.193	−0.026	0.007	1.085	Yes
Moderated indirect effect: IG → FO → CIP	−0.047	−0.081	−0.011	0.007	—	Yes

CIP, continued insurance preparedness; FO, future orientation; IG, immediate gratification; RD, relative deprivation,; VIF, variance inflation factor.

The interaction between relative deprivation and immediate gratification had a significant negative association with future orientation (*β* = −0.113, *p* = 0.007). The negative interaction coefficient indicates that the negative association between immediate gratification and future orientation becomes stronger as relative deprivation increases. This result is consistent with the hypothesized moderating role of relative deprivation and therefore supports H2. Finally, the conditional indirect association between immediate gratification and continued insurance preparedness through future orientation varied significantly with relative deprivation (*β* = −0.047, *p* = 0.007), with the negative indirect association becoming stronger at higher levels of relative deprivation. Thus, H3 was supported.

### 5.5 Multi-group analysis

Before conducting the multigroup analysis, measurement invariance across the urban and rural-hukou groups was assessed using the three-step measurement invariance of composites (MICOM) procedure. The groups were defined according to respondents’ hukou status rather than current place of residence, comprising 192 urban-hukou respondents and 273 rural-hukou respondents. As shown in Table 5, configural invariance and compositional invariance were established for all four constructs, and the differences in composite means and variances fell within the corresponding permutation-based confidence intervals. Thus, full measurement invariance was established across the two groups, supporting subsequent comparisons of the structural relationships.

**Table 5:** Results of 3-step measurement invariance testing using permutation (Urban Hukou—Rural Hukou).

Constructs	Step1	Step2			Step3a (mean)				Step 3b (variance)				Measurement invariance
	Configural invariance	C = 1	5.0% quantile of Cu	Compositional invariance	Differences	2.5%	97.5%	Equal mean value	Differences	2.5%	97.5%	Equal variance	
CIP	Yes	0.998	0.997	Yes	0.102	−0.183	0.185	Yes	−0.001	−0.180	0.182	Yes	Full
FO	Yes	1.000	0.999	Yes	0.078	−0.177	0.196	Yes	0.057	−0.171	0.168	Yes	Full
IG	Yes	1.000	0.996	Yes	−0.096	−0.192	0.190	Yes	−0.007	−0.194	0.210	Yes	Full
RD	Yes	0.997	0.984	Yes	0.010	−0.188	0.174	Yes	−0.036	−0.204	0.186	Yes	Full

CIP, Continued insurance preparedness; FO, Future orientation; IG, Immediate gratification; RD, Relative deprivation.

The multigroup results are presented in Table 6. The indirect association between immediate gratification and continued insurance preparedness through future orientation was stronger in the rural-hukou group (*β* = −0.246) than in the urban-hukou group (*β* = −0.112), with a significant between-group difference before adjustment for multiple comparisons (*Δβ* = 0.134, two-sided *p* = 0.020). The remaining between-group differences, including the associations from immediate gratification to future orientation, immediate gratification to continued insurance preparedness, and future orientation to continued insurance preparedness, as well as the moderating and moderated indirect associations involving relative deprivation, were not statistically significant (all *p* > 0.05). After adjustment for multiple comparisons, the difference in the indirect association was no longer statistically significant (adjusted *p* = 0.120), while the overall pattern continued to suggest a potentially stronger indirect association among rural-hukou respondents.

**Table 6:** Multigroup comparison of structural relationships across urban and rural-hukou groups.

Path/effect	Urban hukou *β*	Rural hukou *β*	*Δβ* (Urban − Rural)	Two-sided *p*	Holm-adjusted *p*
IG → FO	−0.350	−0.494	0.144	0.069	0.345
IG → CIP	−0.025	−0.065	0.040	0.725	0.854
FO → CIP	0.320	0.497	−0.178	0.080	0.345
IG → FO → CIP	−0.112	−0.246	0.134	0.020	0.120
RD × IG → FO	−0.199	−0.069	−0.129	0.127	0.381
RD × IG → FO → CIP	−0.063	−0.034	−0.029	0.427	0.854

CIP, Continued insurance preparedness; FO, Future orientation; IG, Immediate gratification; RD, Relative deprivation.

## 6 Discussion

### 6.1 Main conclusions

Across the two studies, this research examined complementary stages of the proposed psychological process linking immediate gratification, future orientation, and continued insurance preparedness. Study 1 showed that immediate-gratification framing was associated with differences in future orientation, whereas Study 2 examined the relationship between future orientation and continued insurance preparedness within the broader structural model. The results indicate that continued insurance preparedness is a forward-looking psychological state that is closely associated with future-oriented cognition. In Study 2, immediate gratification showed a significant indirect association with continued insurance preparedness through future orientation, whereas its direct association with preparedness was not statistically significant. This pattern highlights future orientation as an important psychological link between present-focused preferences and preparedness for continued insurance participation. This is consistent with the core proposition of SST that perceptions of future time shape goal orientation, such that individuals who maintain attention to future time are more likely to prioritize long-term goals over immediate rewards (18, 19). It also aligns with prior evidence highlighting the central role of future orientation in savings behavior, retirement preparation, and insurance decision-making (25, 28, 41).

With respect to the underlying psychological process, the two studies provide complementary evidence for the importance of future-oriented cognition in linking present-focused preferences with continued insurance preparedness. Study 1 showed that immediate-gratification framing produced significant differences in future orientation, while Study 2 showed that stronger immediate gratification was associated with lower future orientation and that stronger future orientation was associated with greater continued insurance preparedness. Immediate gratification emphasizes present pleasure and immediate rewards and is associated with less attention to future consequences and the protective value of medical insurance. In contrast, individuals with a stronger future orientation are more likely to incorporate future medical needs and insurance coverage into current planning. The significant indirect association observed in Study 2 is consistent with this theoretically proposed process and complements existing evidence showing that immediate gratification and time preferences are related to the evaluation of delayed benefits (20, 42, 43), while future orientation is closely related to long-term financial preparation and insurance decision-making (3, 44, 45). These findings identify future orientation as a theoretically meaningful psychological link between present-focused preferences and preparedness for continued insurance participation.

Further analyses reveal important contextual and group differences in these relationships. The association between immediate gratification and future orientation varied with relative deprivation: the negative relationship was stronger at higher levels of relative deprivation. Correspondingly, the conditional indirect association between immediate gratification and continued insurance preparedness through future orientation also became stronger as relative deprivation increased. This finding resonates with existing evidence linking relative deprivation with stronger short-term orientation and weaker long-term planning (23, 46, 47). Moreover, the multi-group analysis showed a stronger indirect association between immediate gratification and continued insurance preparedness through future orientation among rural-hukou respondents, although the between-group difference did not remain statistically significant after adjustment for multiple comparisons. Although the negative association between immediate gratification and future orientation was numerically stronger in the rural group, the between-group difference did not reach statistical significance. The observed urban–rural difference appears to be concentrated in the indirect association involving future orientation, rather than representing a uniform difference across all relationships in the model. These results align with prior studies documenting structural disparities between urban and rural populations in terms of risk management capacity, institutional accessibility, and long-term planning resources (8, 9, 11).

### 6.2 Theoretical implications

At the theoretical level, this study first extends the explanatory scope of SST to the domain of social security and medical insurance decision-making. Prior research has predominantly applied SST to aging-related decisions, health behaviors, or emotional goal selection, emphasizing how perceptions of future time shape goal orientation (18, 19). Medical insurance provides a useful institutional context for extending this perspective because enrollment requires individuals to make decisions within recurring contribution and coverage cycles while considering uncertain future medical needs. Continued insurance preparedness should therefore be understood not as a realized behavior or a simple delayed-reward outcome, but as a forward-looking psychological state reflecting readiness to maintain coverage and prepare for future insurance-related expenditures. By incorporating future orientation into the relationship between immediate gratification and continued insurance preparedness, this study extends SST to explain how differences in temporal focus are related to psychological readiness for longer-term security planning.

Second, this study deepens understanding of the psychological process connecting short-term orientation with long-term preparatory decision-making within the literature on immediate gratification and time preferences. Existing research has linked present-focused preferences to lower weighting of delayed benefits, with implications for behaviors such as saving, health management, and insurance choice (20, 25, 43). However, this line of research has largely focused on the direct relationship between present-focused preferences and behavioral outcomes, with limited attention to the role of long-term preparatory psychological states. The present findings add to this literature by distinguishing the direct association with insurance preparedness from the indirect association involving future orientation. In particular, the significant indirect association highlights the theoretical relevance of future-oriented cognition for understanding how present-focused preferences are connected with readiness for longer-term security planning. This perspective offers a more differentiated account of the role of intertemporal cognition in insurance-related preparedness.

Third, this study incorporates relative deprivation into the relationship between immediate gratification and future orientation, thereby enriching the theoretical integration of social comparison and long-term decision-making research. Prior studies have indicated that relative deprivation is associated with stronger short-term orientation and impulsive behavior through negative emotions, perceived unfairness, and compensatory emotional needs (21–23). However, this literature has primarily focused on consumption or risk-taking behaviors. The present study extends this line of research by showing that the negative association between immediate gratification and future orientation is stronger under higher relative deprivation and that the corresponding conditional indirect association with continued insurance preparedness also increases in magnitude. These findings connect subjective social comparison with intertemporal cognition in the context of insurance preparedness and suggest an important boundary condition for the relationship between present-focused preferences and longer-term planning. Moreover, the multi-group analysis showed a numerically stronger indirect association through future orientation in the rural-hukou group, although the between-group difference did not remain statistically significant after adjustment for multiple comparisons. This pattern provides a tentative basis for further examining urban–rural heterogeneity in insurance-related preparedness and complements discussions in the urban–rural disparity literature regarding inequalities in risk management capacity and long-term planning resources (9, 11).

### 6.3 Practical implications

The findings offer practical implications for how enrollment support may be designed and evaluated under the basic medical insurance for urban and rural residents, although their applicability across different regional contexts requires further examination. Because participation occurs within recurring contribution and coverage cycles, future interventions could examine whether communication that makes the temporal link between current contributions, subsequent coverage, future medical needs, and continuity of protection more explicit can strengthen residents’ preparedness for continued participation. However, such psychological and communication-based approaches should be considered within the institutional realities of China’s resident medical insurance system, including recurring contribution requirements, subsidy arrangements, household financial constraints, employment instability, and unequal access to healthcare and insurance services. Communication strategies should therefore complement, rather than substitute for, financial and institutional support.

A related implication concerns the social-comparison context in which insurance decisions are made. Communication that strongly emphasizes regional, group, or benefit differences may shape residents’ perceptions of disadvantage and influence how they evaluate long-term protection. Future interventions could therefore test whether messages centered on personal coverage continuity, household risk protection, and future medical needs are more effective than communication relying heavily on comparative cues, particularly among individuals experiencing stronger relative deprivation. At the same time, the findings do not support assuming uniform responses across urban and rural-hukou groups. Possible group differences should be established empirically before differentiated communication strategies are adopted, and any targeted approach should take account of differences in economic conditions, insurance access, and local implementation contexts.

Finally, the practical value of these approaches depends on whether changes in psychological preparedness translate into sustained participation under real enrollment conditions. Future field studies could compare future-oriented communication, reminder strategies, subsidy-based incentives, and combinations of these approaches to determine which forms of support are most effective for different groups and enrollment contexts. Such evaluations should extend beyond self-reported preparedness and incorporate subsequent renewal, payment continuity, and enrollment stability, ideally through longitudinal follow-up or administrative records. This would help distinguish circumstances in which communication-based support is sufficient from those in which affordability, access, or other institutional barriers require stronger financial or policy responses, thereby linking psychological insights more directly to the practical operation of the medical insurance system.

### 6.4 Limitations and future directions

Although this study strengthens the robustness of its findings by combining experimental and survey-based designs, several limitations warrant further investigation.

First, the generalizability of the findings is constrained by the sampling contexts of both studies. Study 1 relied exclusively on undergraduate students, who are relatively homogeneous in age, living conditions, income sources, and medical insurance experience. Whether the observed framing effects extend to middle-aged and older adults, low-income individuals, flexibly employed workers, or other insured populations therefore requires further examination. Study 2, meanwhile, was conducted primarily in Guangxi Province, where regional socioeconomic conditions, population mobility, healthcare resources, and local insurance implementation may differ from those in other parts of China. These population and regional features define important boundary conditions for the present findings. Future research should replicate the experimental design with more heterogeneous community samples and extend the survey to multiple provinces to examine whether the observed relationships remain consistent across age, income, employment status, insurance experience, and regional contexts.

Second, Study 2 measured the focal constructs within the same survey wave, which limits the ability to examine their temporal ordering. Because all focal constructs were self-reported by the same respondents, some common method variance may remain even though the statistical assessments did not indicate a substantial CMB problem. Although Study 1 provides experimental evidence regarding the relationship between immediate-gratification framing and future orientation, the indirect relationship examined in Study 2 is based on cross-sectional data. As cross-sectional mediation estimates may not fully capture psychological processes that unfold over time (48), future research could employ multi-wave or multi-source designs to examine the temporal sequence among immediate gratification, future orientation, and continued insurance preparedness more directly. Such designs would also help clarify how changes in future-oriented cognition correspond with subsequent changes in insurance preparedness.

Third, continued insurance preparedness in this study represents self-reported psychological readiness rather than subsequently observed insurance behavior. Although respondents had current or prior experience with the basic medical insurance for urban and rural residents, the survey did not separately identify current versus former enrollment, responsibility for household contributions, or prior interruption and re-enrollment histories. Future studies could link preparedness measures with contribution records, administrative enrollment histories, or subsequent survey waves to examine whether and under what conditions psychological readiness is reflected in actual renewal, payment continuity, and enrollment stability. This would extend the present focus on preparedness to the behavioral continuity of medical insurance participation.

## Data Availability

The raw data supporting the conclusions of this article will be made available by the authors, without undue reservation.
