# Supplementary material for: Immediate gratification and continued medical insurance preparedness: the roles of future orientation and relative deprivation
Source: Front Public Health. 2026 Oct 19;14:1987239. doi: 10.3389/fpubh.2026.1987239 (PMC13724526; doi:10.3389/fpubh.2026.1987239)
Supplement: Supplementary file 1 [file Table_1.docx]

**城乡居民基本医疗保险决策研究：中英文问卷实施包
Medical Insurance Decision-Making Study: Bilingual Questionnaire Package**

*配套手稿 / Related manuscript:
Immediate gratification and continued medical insurance preparedness: the roles of future orientation and relative deprivation*

# **A. 调查人员实施说明 / Researcher Administration Guide**

**本部分仅供研究团队使用，不向受试者展示。受试者页面中不应出现“高/低即时满足”“高/低相对剥夺”、构念名称、反向计分标记或研究假设。 *This section is for the research team only. Participant-facing materials should not display condition labels, construct names, reverse-scoring markers, or hypotheses.***

## **A1. Study 1：2×2 情景实验 / Study 1: 2×2 Scenario Experiment**

- 目标样本：年满18岁的高校在校生。 *Target sample: currently enrolled university students aged 18 years or older.*
- 实验设计：2（即时满足：高/低）×2（相对剥夺：高/低）组间设计；四组按1:1:1:1随机分配。 *Design: 2 (immediate gratification: high/low) × 2 (relative deprivation: high/low) between-subjects design, randomized 1:1:1:1.*
- 每位受试者只能看到一个完整情景版本；不得先呈现“即时满足材料”再呈现“相对剥夺材料”。 *Each participant must see only one integrated scenario version; do not present the immediate-gratification and relative-deprivation manipulations as separate materials.*
- 建议流程：卷首语 → 知情同意 → 筛选 → 随机呈现一个情景 → 未来取向（主要因变量）→ 操控检验（即时满足、相对剥夺）→ 注意力题 → 人口学信息 → 结束说明。 *Recommended flow: introduction → consent → screening → one randomized scenario → future orientation (primary outcome) → manipulation checks (immediate gratification and relative deprivation) → attention check → demographics → debrief.*
- 为减少需求特征，建议先测量未来取向，再做操控检验。 *To reduce demand characteristics, assess future orientation before manipulation checks.*
- 四个情景版本应使用同一版式、相同阅读时长提示和相同题目顺序。调查员不得解释情景含义或暗示“正确”反应。 *Use the same layout, reading instructions, and item order across all four conditions. Administrators must not explain the manipulation or imply a “correct” response.*
- 纸笔实施可预先按随机序列装订A–D四种版本；线上实施使用平台随机器，将符合条件者等概率分流至A–D。 *For paper administration, prepare A–D packets using a pre-generated random sequence. For online administration, use the platform randomizer to route eligible participants equally to A–D.*

## **A2. Study 1 条件编码 / Study 1 Condition Coding**

| **版本** | **IG编码** | **RD编码** | **内部含义** | **受试者界面** |
| --- | --- | --- | --- | --- |
| A | 1 | 1 | 高IG × 高RD | 仅显示“情境材料” |
| B | 1 | -1 | 高IG × 低RD | 仅显示“情境材料” |
| C | -1 | 1 | 低IG × 高RD | 仅显示“情境材料” |
| D | -1 | -1 | 低IG × 低RD | 仅显示“情境材料” |

## **A3. Study 1 数据质量与排除规则 / Study 1 Data Quality and Exclusion Rules**

- 在正式收集前预先确定排除规则，并在四个实验条件中一致执行。 *Pre-specify exclusion rules before data collection and apply them identically across all four conditions.*
- 排除：未同意参与、未满18岁、非高校在校生、问卷不完整、注意力题未按指定选项作答、明显重复提交或明显不遵守实验要求。 *Exclusions: no consent, under 18, not currently enrolled at a university, incomplete responses, failed instructed-response attention check, clear duplicate submissions, or clear noncompliance with experimental instructions.*
- 若使用“完成时间过短”标准，应根据预测试预先确定最低合理时长，不要在看到结果后再选择阈值。 *If a minimum completion-time rule is used, define it from the pilot before main data collection rather than choosing a threshold after inspecting results.*
- 操控检验不建议作为“是否保留个案”的唯一依据；其主要用途是检验实验操控是否在组水平上生效。 *Manipulation-check scores should not be the sole basis for excluding individual cases; their main purpose is to verify condition-level manipulation effectiveness.*

## **A4. Study 2：自然情境问卷 / Study 2: Naturalistic Survey**

- 目标样本：年满18岁、目前在广西居住，并且本人目前或过去曾参加城乡居民基本医疗保险的居民。 *Target sample: adults aged 18 or older who currently reside in Guangxi and have current or prior experience with the Basic Medical Insurance for Urban and Rural Residents.*
- 建议流程：卷首语 → 知情同意 → 筛选 → 即时满足 → 未来取向 → 注意力题1 → 相对剥夺 → 持续参保准备 → 注意力题2 → 人口学与参保信息 → 结束说明。 *Recommended flow: introduction → consent → screening → immediate gratification → future orientation → attention check 1 → relative deprivation → continued insurance preparedness → attention check 2 → demographics/enrollment information → closing.*
- 线上问卷建议启用匿名的重复作答限制；不要收集姓名、身份证号、手机号等与研究目的无关的可识别信息。 *For online administration, use anonymous duplicate-response controls where available and avoid collecting names, ID numbers, phone numbers, or other identifiers not needed for the research.*

## **A5. 量表编码表 / Scale Coding Key**

**所有核心题项均采用7点Likert量表。反向计分仅在数据处理中执行：反向题得分 = 8 − 原始得分。 *All core items use a 7-point Likert scale. Reverse scoring is performed only during data processing: reversed score = 8 − raw score.***

| **构念** | **变量代码** | **题数** | **反向题** |  |
| --- | --- | --- | --- | --- |
| 未来取向 / Future orientation | FO1–FO4 | 4 | FO3, FO4 |  |
| 即时满足 / Immediate gratification | IG1–IG7 | 7 | 无 / None |  |
| 相对剥夺 / Relative deprivation | RD1–RD5 | 5 | RD2, RD4 |  |
| 持续参保准备 / Continued insurance preparedness | CIP1–CIP4 | 4 | 无 / None |  |
| **【研究人员用 / Researcher only】 量表来源按手稿保持：即时满足基于 Joireman et al.; 未来取向基于 Hershey & Mowen; 相对剥夺基于 Callan et al.; 持续参保准备结合 Mamun et al. 与 Hershey & Mowen 进行情境化。** *Scale provenance follows the manuscript: immediate gratification (Joireman et al.); future orientation (Hershey & Mowen); relative deprivation (Callan et al.); continued insurance preparedness contextualized from Mamun et al. and Hershey & Mowen.* | | | | |

# **B. Study 1 发放母版 / Study 1 Deployment Master**

# **Study 1 受试者问卷 / Study 1 Participant Questionnaire**

尊敬的参与者：
您好！我们正在开展一项关于医疗保障相关信息、判断与决策的学术研究。本问卷没有标准答案，我们关注的是您在特定情境或日常生活中的真实看法。请独立完成问卷，不与他人讨论。您的回答仅用于学术研究和统计分析。 *Dear Participant,
We are conducting an academic study on how people perceive and make judgments about information and decisions related to medical protection. There are no correct or incorrect answers. We are interested in your genuine views in the scenario or in everyday life. Please complete the questionnaire independently without discussing your answers with others. Your responses will be used only for academic research and statistical analysis.*

预计完成时间约10分钟。 *The questionnaire takes approximately 10 minutes to complete.*

## **知情同意 / Informed Consent**

请在继续前仔细阅读以下说明： *Please read the following information carefully before continuing:*

- 我已了解本研究的大致目的与填写流程，知晓参与完全自愿。 *I understand the general purpose and procedure of the study and that participation is entirely voluntary.*
- 本研究为低风险学术调查；部分题目涉及医疗保障、未来规划或与他人的比较，可能引起轻微不适。如感到不适，我可以停止作答。 *This is a low-risk academic study. Some questions concern medical protection, future planning, or comparisons with others and may cause mild discomfort. I may stop at any time if I feel uncomfortable.*
- 我可以在提交问卷前随时退出，不需要说明理由，也不会受到任何不利影响。 *I may withdraw at any time before submitting the questionnaire without giving a reason and without any penalty.*
- 问卷采用匿名方式，不收集姓名、身份证号等直接身份信息。数据仅用于学术研究和统计分析。 *The questionnaire is anonymous and does not collect direct identifiers such as names or national ID numbers. Data will be used only for academic research and statistical analysis.*
- 由于数据匿名，问卷提交后研究团队通常无法定位并单独删除某一份答卷。 *Because responses are anonymous, after submission the research team will generally be unable to identify and delete a specific individual response.*
- 如对研究有疑问，可联系研究团队：	。 *For questions about the study, you may contact the research team at	.*

**C1. 您是否同意参加本研究？** *Do you agree to participate in this study?*

□ 我已阅读并同意，自愿参加 */ I have read the information above and voluntarily agree to participate*

□ 我不同意参加 */ I do not agree to participate*

**【研究人员用 / Researcher only】 选择“不同意”→立即结束问卷，不进入筛选题。** *Selecting “I do not agree” should immediately terminate the survey before screening.*

## **受试者筛选 / Eligibility Screening**

**S1. 您是否年满18周岁？** *Are you 18 years of age or older?*

□ 是 */ Yes*	□ 否 */ No*

**S2. 您目前是否为高校在校生（专科、本科或研究生均可）？** *Are you currently enrolled at a college or university (including junior college, undergraduate, or postgraduate study)?*

□ 是 */ Yes*	□ 否 */ No*

**【研究人员用 / Researcher only】 纳入条件：S1=是 且 S2=是。其他情况→结束页。** *Eligibility: S1 = Yes AND S2 = Yes. Otherwise route to the termination page.*

## **第1部分：情境材料（随机呈现一个版本） / Part 1: Scenario (Randomly Present One Version)**

**【研究人员用 / Researcher only】 以下A–D仅用于研究者建问卷/装订版本。受试者只能看到其中一个，并且页面上不要显示A–D对应的高/低条件含义。** *A–D are for survey programming/packet preparation only. Each participant must see only one version, and the high/low condition meaning must not be displayed.*

### **版本 A（研究者内部：高即时满足 × 高相对剥夺 / High IG × High RD） / Version A (researcher coding only)**

请设想以下情境发生在你身上。以下材料为研究构造的假设情境，不代表任何真实地区的医保政策。 *Please imagine that the following situation happens to you. This is a hypothetical scenario created for research purposes and does not describe the actual medical insurance policy of any real region.*

新一年度城乡居民基本医疗保险集中参保/缴费期临近。学校提醒同学们留意参保安排。与此同时，你也在安排这个月有限的生活费，需要在日常消费和一些面向未来的支出之间做取舍。 *The annual enrollment/contribution period for the Basic Medical Insurance for Urban and Rural Residents is approaching, and your university has reminded students to pay attention to their enrollment arrangements. At the same time, you are planning how to use your limited living allowance this month and need to balance everyday spending with expenses that may benefit you in the future.*

最近课程和生活节奏较快，你希望能让自己轻松一些。聚餐、看电影、短途出行或买一件喜欢的东西，都能很快带来放松和愉快；相比之下，医疗保险等长期保障在短期内通常看不到明显回报，未来是否会发生医疗支出也不确定。你因此更容易把注意力放在眼下可见的效果上，觉得先照顾当前的需要、未来的事情以后再处理也来得及。 *Recently, your academic and daily schedule has been busy, and you would like to make life feel a little easier. Having a meal with friends, watching a movie, taking a short trip, or buying something you like can quickly bring relaxation and enjoyment. By comparison, long-term protection such as medical insurance usually brings no visible return in the short term, and it is uncertain whether you will face medical expenses in the future. You therefore find it easier to focus on benefits that can be felt now and to think that current needs can come first while future issues can be dealt with later.*

就在你考虑这些问题时，你看到一份关于不同地区医保待遇的说明。与你年龄和家庭经济条件相近、缴费水平也基本相同的同龄人中，一些其他地区的参保者住院报销比例更高、年度报销限额更高，可报销药品和项目范围更广，异地就医结算也更方便；你所在地区在这些方面相对较低或受限，办理手续也更繁琐。也就是说，在投入大致相同的情况下，你能获得的医疗保障明显少于这些相似的同龄人。 *While considering these issues, you read an information notice comparing medical insurance benefits across regions. Among people of a similar age and family economic background who pay roughly the same contribution, some peers in other regions have higher inpatient reimbursement rates, higher annual reimbursement limits, a broader range of reimbursable medicines and services, and more convenient cross-region settlement procedures. In your region, these benefits are relatively lower or more restricted, and the procedures are more complicated. In other words, despite making a similar contribution, the medical protection available to you is clearly less favorable than that available to these comparable peers.*

请保持上述情境，想象自己正处在这种情况下。接下来的问题没有正确或错误答案，请根据你在该情境下最真实的感受作答。 *Please keep the situation above in mind and imagine that you are personally experiencing it. There are no right or wrong answers to the following questions. Please respond according to how you genuinely feel in this situation.*

— 研究者分隔线 / Researcher separator —

### **版本 B（研究者内部：高即时满足 × 低相对剥夺 / High IG × Low RD） / Version B (researcher coding only)**

请设想以下情境发生在你身上。以下材料为研究构造的假设情境，不代表任何真实地区的医保政策。 *Please imagine that the following situation happens to you. This is a hypothetical scenario created for research purposes and does not describe the actual medical insurance policy of any real region.*

新一年度城乡居民基本医疗保险集中参保/缴费期临近。学校提醒同学们留意参保安排。与此同时，你也在安排这个月有限的生活费，需要在日常消费和一些面向未来的支出之间做取舍。 *The annual enrollment/contribution period for the Basic Medical Insurance for Urban and Rural Residents is approaching, and your university has reminded students to pay attention to their enrollment arrangements. At the same time, you are planning how to use your limited living allowance this month and need to balance everyday spending with expenses that may benefit you in the future.*

最近课程和生活节奏较快，你希望能让自己轻松一些。聚餐、看电影、短途出行或买一件喜欢的东西，都能很快带来放松和愉快；相比之下，医疗保险等长期保障在短期内通常看不到明显回报，未来是否会发生医疗支出也不确定。你因此更容易把注意力放在眼下可见的效果上，觉得先照顾当前的需要、未来的事情以后再处理也来得及。 *Recently, your academic and daily schedule has been busy, and you would like to make life feel a little easier. Having a meal with friends, watching a movie, taking a short trip, or buying something you like can quickly bring relaxation and enjoyment. By comparison, long-term protection such as medical insurance usually brings no visible return in the short term, and it is uncertain whether you will face medical expenses in the future. You therefore find it easier to focus on benefits that can be felt now and to think that current needs can come first while future issues can be dealt with later.*

就在你考虑这些问题时，你看到一份关于不同地区医保待遇的说明。与你年龄和家庭经济条件相近、缴费水平也基本相同的同龄人相比，你所在地区的住院报销比例、年度报销限额、可报销药品和项目范围，以及异地就医结算便利程度都大体相近，办理手续所需材料和等待时间也差别不大，各地区在总体保障水平上没有明显差异。也就是说，在投入大致相同的情况下，你与这些相似同龄人能够获得的医疗保障总体相当。 *While considering these issues, you read an information notice comparing medical insurance benefits across regions. Among people of a similar age and family economic background who pay roughly the same contribution, the inpatient reimbursement rates, annual reimbursement limits, range of reimbursable medicines and services, and convenience of cross-region settlement in your region are generally similar to those in other regions. The required documents and waiting times are also broadly comparable, with no clear overall advantage or disadvantage. In other words, with a similar contribution, the medical protection available to you is generally comparable to that available to these similar peers.*

请保持上述情境，想象自己正处在这种情况下。接下来的问题没有正确或错误答案，请根据你在该情境下最真实的感受作答。 *Please keep the situation above in mind and imagine that you are personally experiencing it. There are no right or wrong answers to the following questions. Please respond according to how you genuinely feel in this situation.*

— 研究者分隔线 / Researcher separator —

### **版本 C（研究者内部：低即时满足 × 高相对剥夺 / Low IG × High RD） / Version C (researcher coding only)**

请设想以下情境发生在你身上。以下材料为研究构造的假设情境，不代表任何真实地区的医保政策。 *Please imagine that the following situation happens to you. This is a hypothetical scenario created for research purposes and does not describe the actual medical insurance policy of any real region.*

新一年度城乡居民基本医疗保险集中参保/缴费期临近。学校提醒同学们留意参保安排。与此同时，你也在安排这个月有限的生活费，需要在日常消费和一些面向未来的支出之间做取舍。 *The annual enrollment/contribution period for the Basic Medical Insurance for Urban and Rural Residents is approaching, and your university has reminded students to pay attention to their enrollment arrangements. At the same time, you are planning how to use your limited living allowance this month and need to balance everyday spending with expenses that may benefit you in the future.*

最近课程和生活节奏较快，你也在认真考虑怎样安排有限的生活费。聚餐、看电影、短途出行或买一件喜欢的东西虽然能带来即时放松，但这种满足通常比较短暂；相比之下，医疗保险等长期保障虽然短期内看不到明显回报，却可能在未来出现健康支出时发挥作用。你因此更容易把注意力放在未来可能的需要上，觉得现在做一些准备能让之后的生活更稳定、更从容。 *Recently, your academic and daily schedule has been busy, and you have been thinking carefully about how to use your limited living allowance. Having a meal with friends, watching a movie, taking a short trip, or buying something you like can bring immediate relaxation, but that satisfaction is usually short-lived. By comparison, long-term protection such as medical insurance may bring no visible return in the short term, yet it could matter if health-related expenses arise in the future. You therefore find it easier to focus on possible future needs and to think that making some preparations now can make your future life more stable and manageable.*

就在你考虑这些问题时，你看到一份关于不同地区医保待遇的说明。与你年龄和家庭经济条件相近、缴费水平也基本相同的同龄人中，一些其他地区的参保者住院报销比例更高、年度报销限额更高，可报销药品和项目范围更广，异地就医结算也更方便；你所在地区在这些方面相对较低或受限，办理手续也更繁琐。也就是说，在投入大致相同的情况下，你能获得的医疗保障明显少于这些相似的同龄人。 *While considering these issues, you read an information notice comparing medical insurance benefits across regions. Among people of a similar age and family economic background who pay roughly the same contribution, some peers in other regions have higher inpatient reimbursement rates, higher annual reimbursement limits, a broader range of reimbursable medicines and services, and more convenient cross-region settlement procedures. In your region, these benefits are relatively lower or more restricted, and the procedures are more complicated. In other words, despite making a similar contribution, the medical protection available to you is clearly less favorable than that available to these comparable peers.*

请保持上述情境，想象自己正处在这种情况下。接下来的问题没有正确或错误答案，请根据你在该情境下最真实的感受作答。 *Please keep the situation above in mind and imagine that you are personally experiencing it. There are no right or wrong answers to the following questions. Please respond according to how you genuinely feel in this situation.*

— 研究者分隔线 / Researcher separator —

### **版本 D（研究者内部：低即时满足 × 低相对剥夺 / Low IG × Low RD） / Version D (researcher coding only)**

请设想以下情境发生在你身上。以下材料为研究构造的假设情境，不代表任何真实地区的医保政策。 *Please imagine that the following situation happens to you. This is a hypothetical scenario created for research purposes and does not describe the actual medical insurance policy of any real region.*

新一年度城乡居民基本医疗保险集中参保/缴费期临近。学校提醒同学们留意参保安排。与此同时，你也在安排这个月有限的生活费，需要在日常消费和一些面向未来的支出之间做取舍。 *The annual enrollment/contribution period for the Basic Medical Insurance for Urban and Rural Residents is approaching, and your university has reminded students to pay attention to their enrollment arrangements. At the same time, you are planning how to use your limited living allowance this month and need to balance everyday spending with expenses that may benefit you in the future.*

最近课程和生活节奏较快，你也在认真考虑怎样安排有限的生活费。聚餐、看电影、短途出行或买一件喜欢的东西虽然能带来即时放松，但这种满足通常比较短暂；相比之下，医疗保险等长期保障虽然短期内看不到明显回报，却可能在未来出现健康支出时发挥作用。你因此更容易把注意力放在未来可能的需要上，觉得现在做一些准备能让之后的生活更稳定、更从容。 *Recently, your academic and daily schedule has been busy, and you have been thinking carefully about how to use your limited living allowance. Having a meal with friends, watching a movie, taking a short trip, or buying something you like can bring immediate relaxation, but that satisfaction is usually short-lived. By comparison, long-term protection such as medical insurance may bring no visible return in the short term, yet it could matter if health-related expenses arise in the future. You therefore find it easier to focus on possible future needs and to think that making some preparations now can make your future life more stable and manageable.*

就在你考虑这些问题时，你看到一份关于不同地区医保待遇的说明。与你年龄和家庭经济条件相近、缴费水平也基本相同的同龄人相比，你所在地区的住院报销比例、年度报销限额、可报销药品和项目范围，以及异地就医结算便利程度都大体相近，办理手续所需材料和等待时间也差别不大，各地区在总体保障水平上没有明显差异。也就是说，在投入大致相同的情况下，你与这些相似同龄人能够获得的医疗保障总体相当。 *While considering these issues, you read an information notice comparing medical insurance benefits across regions. Among people of a similar age and family economic background who pay roughly the same contribution, the inpatient reimbursement rates, annual reimbursement limits, range of reimbursable medicines and services, and convenience of cross-region settlement in your region are generally similar to those in other regions. The required documents and waiting times are also broadly comparable, with no clear overall advantage or disadvantage. In other words, with a similar contribution, the medical protection available to you is generally comparable to that available to these similar peers.*

请保持上述情境，想象自己正处在这种情况下。接下来的问题没有正确或错误答案，请根据你在该情境下最真实的感受作答。 *Please keep the situation above in mind and imagine that you are personally experiencing it. There are no right or wrong answers to the following questions. Please respond according to how you genuinely feel in this situation.*

**【研究人员用 / Researcher only】 线上问卷：随机器之后直接跳转到第2部分；不要让受试者继续滚动看到其他三个情境。纸笔问卷：A–D应分别装订，只保留对应情境。** *Online: after randomization, route directly to Part 2 and prevent scrolling into the other scenarios. Paper: prepare separate A–D packets containing only the assigned scenario.*

## **第2部分：您在情境中的想法 / Part 2: Your Thoughts in the Scenario**

请继续保持刚才的情境，根据您在该情境下此刻的真实感受作答。 *Please keep the scenario in mind and respond according to how you genuinely feel in that situation.*

**1 = 完全不同意	2 = 不同意	3 = 比较不同意	4 = 既不同意也不反对	5 = 比较同意	6 = 同意	7 = 完全同意** *1 = Strongly disagree	2 = Disagree	3 = Somewhat disagree	4 = Neither agree nor disagree	5 = Somewhat agree	6 = Agree	7 = Strongly agree*

| **题项 / Item** | **作答 / Response** |
| --- | --- |
| FO1. 我会主动思考未来的医疗需求，并在当前决策中考虑未来可能发生的健康相关事件。 *I would actively think about my future medical needs and take possible health-related events into consideration when making decisions now.* | □1 □2 □3 □4 □5 □6 □7 |
| FO2. 我愿意为未来可能的医疗风险提前规划，并采取行动保持稳定的参保状态。 *I would be willing to plan ahead for possible future medical risks and take action to maintain stable insurance coverage.* | □1 □2 □3 □4 □5 □6 □7 |
| FO3. 我觉得未来过于不确定，因此难以根据长期医疗保障需求作判断。 *I feel that the future is too uncertain to make decisions based on long-term medical protection needs.* | □1 □2 □3 □4 □5 □6 □7 |
| FO4. 我更倾向于关注医保缴费的眼前成本，而不是其长期收益。 *I tend to focus more on the immediate costs of medical insurance contributions than on their long-term benefits.* | □1 □2 □3 □4 □5 □6 □7 |

## **第3部分：对刚才情境的感受 / Part 3: Reactions to the Scenario**

下面的问题仍然针对刚才阅读的同一个情境。 *The following questions refer to the same scenario you have just read.*

**1 = 完全不同意	2 = 不同意	3 = 比较不同意	4 = 既不同意也不反对	5 = 比较同意	6 = 同意	7 = 完全同意** *1 = Strongly disagree	2 = Disagree	3 = Somewhat disagree	4 = Neither agree nor disagree	5 = Somewhat agree	6 = Agree	7 = Strongly agree*

| **题项 / Item** | **作答 / Response** |
| --- | --- |
| IG1. 在刚才的情境中，我更希望做决定时能看到比较快的效果，而不是等很久才有结果。 *In the scenario, I would prefer decisions that show results sooner rather than much later.* | □1 □2 □3 □4 □5 □6 □7 |
| IG2. 在刚才的情境中，我更倾向于优先考虑短期能带来好处的事情。 *In the scenario, I would tend to prioritize things that bring short-term benefits.* | □1 □2 □3 □4 □5 □6 □7 |
| IG3. 在刚才的情境中，我更希望选择那些能马上让我轻松一点的选项。 *In the scenario, I would prefer options that could make me feel better right away.* | □1 □2 □3 □4 □5 □6 □7 |
| IG4. 在刚才的情境中，我不太想为未来的问题操心太多，因为我觉得它们通常可以之后再处理。 *In the scenario, I would prefer not to worry too much about future problems because they could usually be dealt with later.* | □1 □2 □3 □4 □5 □6 □7 |
| IG5. 在刚才的情境中，我不希望为了很久以后的事情而放弃当下的小快乐。 *In the scenario, I would prefer not to give up small present pleasures for something far in the future.* | □1 □2 □3 □4 □5 □6 □7 |
| IG6. 在刚才的情境中，我更希望先顾好眼前的需要，未来的问题以后再想。 *In the scenario, I would prefer to take care of immediate needs first and think about future issues later.* | □1 □2 □3 □4 □5 □6 □7 |
| IG7. 在刚才的情境中，近期能看到变化的事情比遥远的结果更有吸引力。 *In the scenario, things with more immediate results would be more appealing to me than distant outcomes.* | □1 □2 □3 □4 □5 □6 □7 |

| **题项 / Item** | **作答 / Response** |
| --- | --- |
| RD1. 当我想到自己能够获得的医疗保障与经济条件相似的人相比时，我会感到自己受到了不公平对待。 *I would feel unfairly treated when I think about the medical insurance benefits available to me compared with people in similar economic circumstances.* | □1 □2 □3 □4 □5 □6 □7 |
| RD2. 与经济条件相似的人相比，我会觉得自己在医疗保障方面更为优越。 *Compared with people in similar economic circumstances, I would feel relatively privileged in terms of medical insurance benefits.* | □1 □2 □3 □4 □5 □6 □7 |
| RD3. 当我看到其他地区或相似的人享受更好的医疗保障时，我会感到不满或委屈。 *I would feel resentful or upset if I saw people in other regions or similar others receiving better medical insurance benefits.* | □1 □2 □3 □4 □5 □6 □7 |
| RD4. 当我比较自己与相似他人的医保待遇时，我会觉得自己的保障其实还不错。 *When comparing my medical insurance benefits with those of similar others, I would feel that my own protection is actually quite good.* | □1 □2 □3 □4 □5 □6 □7 |
| RD5. 当我将自己能够获得的医保待遇与相似他人相比时，我会对目前的保障状况感到不满意。 *I would feel dissatisfied with my current medical insurance benefits when comparing them with what similar others receive.* | □1 □2 □3 □4 □5 □6 □7 |

## **第4部分：作答确认 / Part 4: Response Check**

请按照题目本身的要求作答。 *Please answer according to the instruction in the item itself.*

**1 = 完全不同意	2 = 不同意	3 = 比较不同意	4 = 既不同意也不反对	5 = 比较同意	6 = 同意	7 = 完全同意** *1 = Strongly disagree	2 = Disagree	3 = Somewhat disagree	4 = Neither agree nor disagree	5 = Somewhat agree	6 = Agree	7 = Strongly agree*

| **题项 / Item** | **作答 / Response** |
| --- | --- |
| AC1. 为了确认您正在认真阅读，请在本题选择“4—既不同意也不反对”。 *To confirm that you are reading carefully, please select “4 — Neither agree nor disagree” for this item.* | □1 □2 □3 □4 □5 □6 □7 |

## **第5部分：基本信息 / Part 5: Demographic Information**

**D1. 您的性别是？** *What is your gender?*

□ 男 */ Male*	□ 女 */ Female*	□ 其他/自我描述 */ Other / self-describe*	□ 不愿透露 */ Prefer not to say*

**D2. 您的年龄是？** *What is your age?*

______________________________

**D3. 您目前的年级是？** *What is your current year of study?*

□ 大一 */ Year 1*

□ 大二 */ Year 2*

□ 大三 */ Year 3*

□ 大四及以上 */ Year 4 or above*

□ 研究生 */ Postgraduate*

**D4. 您每月可支配生活费大约是多少？** *What is your approximate monthly living allowance?*

□ 少于1,000元 */ Less than RMB 1,000*

□ 1,000–1,999元 */ RMB 1,000–1,999*

□ 2,000–2,999元 */ RMB 2,000–2,999*

□ 3,000–3,999元 */ RMB 3,000–3,999*

□ 4,000元及以上 */ RMB 4,000 or above*

**D5. 您家庭的户籍类型是？** *What is your family household registration type (hukou)?*

□ 城镇户口 */ Urban*	□ 农村户口 */ Rural*	□ 不确定/其他 */ Not sure / Other*

**D6. 您目前是否参加城乡居民基本医疗保险？** *Are you currently enrolled in the Basic Medical Insurance for Urban and Rural Residents?*

□ 是 */ Yes*	□ 否 */ No*	□ 不确定 */ Not sure*

## **结束说明 / Debrief and Closing**

**感谢您的参与！刚才的医保信息为研究构造的假设情境，不代表任何真实地区的政策。不同参与者可能看到不同版本的情境材料，用于比较人们在不同信息环境下的判断。请暂时不要与尚未参加研究的同学讨论具体情境内容，以免影响后续数据收集。 *Thank you for participating. The medical insurance information in the scenario was hypothetical and does not describe any real regional policy. Different participants may receive different versions so that we can compare judgments under different information conditions. Please avoid discussing the specific scenario with students who have not yet participated, as this could affect later data collection.***

# **C. Study 2 可直接发放问卷 / Study 2 Direct-Distribution Questionnaire**

# **Study 2 受试者问卷 / Study 2 Participant Questionnaire**

尊敬的参与者：
您好！我们正在开展一项关于医疗保障相关信息、判断与决策的学术研究。本问卷没有标准答案，我们关注的是您在特定情境或日常生活中的真实看法。请独立完成问卷，不与他人讨论。您的回答仅用于学术研究和统计分析。 *Dear Participant,
We are conducting an academic study on how people perceive and make judgments about information and decisions related to medical protection. There are no correct or incorrect answers. We are interested in your genuine views in the scenario or in everyday life. Please complete the questionnaire independently without discussing your answers with others. Your responses will be used only for academic research and statistical analysis.*

预计完成时间约10分钟。 *The questionnaire takes approximately 10 minutes to complete.*

## **知情同意 / Informed Consent**

请在继续前仔细阅读以下说明： *Please read the following information carefully before continuing:*

- 我已了解本研究的大致目的与填写流程，知晓参与完全自愿。 *I understand the general purpose and procedure of the study and that participation is entirely voluntary.*
- 本研究为低风险学术调查；部分题目涉及医疗保障、未来规划或与他人的比较，可能引起轻微不适。如感到不适，我可以停止作答。 *This is a low-risk academic study. Some questions concern medical protection, future planning, or comparisons with others and may cause mild discomfort. I may stop at any time if I feel uncomfortable.*
- 我可以在提交问卷前随时退出，不需要说明理由，也不会受到任何不利影响。 *I may withdraw at any time before submitting the questionnaire without giving a reason and without any penalty.*
- 问卷采用匿名方式，不收集姓名、身份证号等直接身份信息。数据仅用于学术研究和统计分析。 *The questionnaire is anonymous and does not collect direct identifiers such as names or national ID numbers. Data will be used only for academic research and statistical analysis.*
- 由于数据匿名，问卷提交后研究团队通常无法定位并单独删除某一份答卷。 *Because responses are anonymous, after submission the research team will generally be unable to identify and delete a specific individual response.*
- 如对研究有疑问，可联系研究团队：	。 *For questions about the study, you may contact the research team at	.*

**C1. 您是否同意参加本研究？** *Do you agree to participate in this study?*

□ 我已阅读并同意，自愿参加 */ I have read the information above and voluntarily agree to participate*

□ 我不同意参加 */ I do not agree to participate*

**【研究人员用 / Researcher only】 线上逻辑：选择“不同意”→立即结束问卷，不进入筛选题。** *Online logic: selecting “I do not agree” should immediately terminate the survey before screening.*

## **受试者筛选 / Eligibility Screening**

**S1. 您是否年满18周岁？** *Are you 18 years of age or older?*

□ 是 */ Yes*	□ 否 */ No*

**S2. 您目前是否在广西壮族自治区居住？** *Do you currently reside in Guangxi Zhuang Autonomous Region?*

□ 是 */ Yes*	□ 否 */ No*

**S3. 您本人目前或过去是否参加过城乡居民基本医疗保险？** *Are you currently enrolled, or have you previously been enrolled, in the Basic Medical Insurance for Urban and Rural Residents?*

□ 是 */ Yes*	□ 否 */ No*	□ 不确定 */ Not sure*

**【研究人员用 / Researcher only】 建议纳入条件：S1=是、S2=是、S3=是。S3用于确保受试者对后续医保待遇与持续参保题目具有基本经验基础。** *Recommended eligibility: S1 = Yes, S2 = Yes, S3 = Yes. S3 helps ensure respondents have sufficient experience to answer later questions about benefits and continued participation.*

## **第1部分：日常看法与倾向 / Part 1: Everyday Views and Tendencies**

以下陈述描述您在日常生活中对短期与长期结果的通常看法。请根据您平时较为稳定的真实倾向作答。 *The following statements describe your usual views of short-term and long-term outcomes in everyday life. Please answer according to your typical, stable tendencies.*

**1 = 完全不同意	2 = 不同意	3 = 比较不同意	4 = 既不同意也不反对	5 = 比较同意	6 = 同意	7 = 完全同意** *1 = Strongly disagree	2 = Disagree	3 = Somewhat disagree	4 = Neither agree nor disagree	5 = Somewhat agree	6 = Agree	7 = Strongly agree*

| **题项 / Item** | **作答 / Response** |
| --- | --- |
| IG1. 一般来说，我更希望自己做决定时能看到比较快的效果，而不是等很久才有结果。 *In general, I prefer making decisions that show results sooner rather than much later.* | □1 □2 □3 □4 □5 □6 □7 |
| IG2. 在选择时，我更倾向于优先考虑短期能带来好处的事情。 *When choosing, I tend to focus more on things that bring short-term benefits.* | □1 □2 □3 □4 □5 □6 □7 |
| IG3. 我更喜欢那些能马上让我轻松一点的选项。 *I prefer options that can make me feel better right away.* | □1 □2 □3 □4 □5 □6 □7 |
| IG4. 我不太想为未来的问题操心太多，因为我觉得它们通常可以之后再处理。 *I prefer not to worry too much about future problems because I feel they can usually be dealt with later.* | □1 □2 □3 □4 □5 □6 □7 |
| IG5. 我不希望为了很久以后的事情而放弃当下的小快乐。 *I prefer not to give up small present pleasures for something far in the future.* | □1 □2 □3 □4 □5 □6 □7 |
| IG6. 我更希望先顾好眼前的需要，未来的问题以后再想。 *I prefer to take care of my immediate needs first and think about future issues later.* | □1 □2 □3 □4 □5 □6 □7 |
| IG7. 对我来说，近期能看到变化的事情比遥远的结果更有吸引力。 *Things with more immediate results are more appealing to me than distant outcomes.* | □1 □2 □3 □4 □5 □6 □7 |

以下陈述涉及您对未来医疗需求和长期保障的通常看法。 *The following statements concern your usual views about future medical needs and long-term protection.*

**1 = 完全不同意	2 = 不同意	3 = 比较不同意	4 = 既不同意也不反对	5 = 比较同意	6 = 同意	7 = 完全同意** *1 = Strongly disagree	2 = Disagree	3 = Somewhat disagree	4 = Neither agree nor disagree	5 = Somewhat agree	6 = Agree	7 = Strongly agree*

| **题项 / Item** | **作答 / Response** |
| --- | --- |
| FO1. 我会主动思考未来的医疗需求，并在当前决策中考虑未来可能发生的健康相关事件。 *I actively think about my future medical needs and take possible health-related events into consideration when making decisions today.* | □1 □2 □3 □4 □5 □6 □7 |
| FO2. 我愿意为未来可能的医疗风险提前规划，并采取行动保持稳定的参保状态。 *I am willing to plan ahead for possible future medical risks and take action to maintain stable insurance coverage.* | □1 □2 □3 □4 □5 □6 □7 |
| FO3. 我觉得未来过于不确定，因此难以根据长期医疗保障需求作判断。 *I feel that the future is too uncertain to make decisions based on long-term medical protection needs.* | □1 □2 □3 □4 □5 □6 □7 |
| FO4. 我更倾向于关注医保缴费的眼前成本，而不是其长期收益。 *I tend to focus more on the immediate costs of medical insurance contributions than on their long-term benefits.* | □1 □2 □3 □4 □5 □6 □7 |

| **题项 / Item** | **作答 / Response** |
| --- | --- |
| AC1. 为了确认您正在认真阅读，请在本题选择“2—不同意”。 *To confirm that you are reading carefully, please select “2 — Disagree” for this item.* | □1 □2 □3 □4 □5 □6 □7 |

## **第2部分：医疗保障比较感受 / Part 2: Feelings About Medical Insurance Comparisons**

以下陈述描述您在日常生活中将自己的医疗保障与经济条件相似的人进行比较时的感受。 *The following statements describe how you feel when comparing your medical insurance protection with that of people in similar economic circumstances.*

**1 = 完全不同意	2 = 不同意	3 = 比较不同意	4 = 既不同意也不反对	5 = 比较同意	6 = 同意	7 = 完全同意** *1 = Strongly disagree	2 = Disagree	3 = Somewhat disagree	4 = Neither agree nor disagree	5 = Somewhat agree	6 = Agree	7 = Strongly agree*

| **题项 / Item** | **作答 / Response** |
| --- | --- |
| RD1. 当我想到自己能够获得的医疗保障与经济条件相似的人相比时，我会感到自己受到了不公平对待。 *I feel unfairly treated when I think about the medical insurance benefits available to me compared with people in similar economic circumstances.* | □1 □2 □3 □4 □5 □6 □7 |
| RD2. 与经济条件相似的人相比，我觉得自己在医疗保障方面更为优越。 *Compared with people in similar economic circumstances, I feel relatively privileged in terms of medical insurance benefits.* | □1 □2 □3 □4 □5 □6 □7 |
| RD3. 当我看到其他地区或相似的人享受更好的医疗保障时，我会感到不满或委屈。 *I feel resentful or upset when I see people in other regions or similar others receiving better medical insurance benefits.* | □1 □2 □3 □4 □5 □6 □7 |
| RD4. 当我比较自己与相似他人的医保待遇时，我会觉得自己的保障其实还不错。 *When comparing my medical insurance benefits with those of similar others, I feel that my own protection is actually quite good.* | □1 □2 □3 □4 □5 □6 □7 |
| RD5. 当我将自己能够获得的医保待遇与相似他人相比时，我会对目前的保障状况感到不满意。 *I feel dissatisfied with my current medical insurance benefits when comparing them with what similar others receive.* | □1 □2 □3 □4 □5 □6 □7 |

## **第3部分：持续参保准备 / Part 3: Continued Insurance Preparedness**

以下陈述涉及您对未来持续参加医疗保险以及为相关支出做准备的程度。 *The following statements concern your preparedness to maintain medical insurance participation and plan for related future expenses.*

**1 = 完全不同意	2 = 不同意	3 = 比较不同意	4 = 既不同意也不反对	5 = 比较同意	6 = 同意	7 = 完全同意** *1 = Strongly disagree	2 = Disagree	3 = Somewhat disagree	4 = Neither agree nor disagree	5 = Somewhat agree	6 = Agree	7 = Strongly agree*

| **题项 / Item** | **作答 / Response** |
| --- | --- |
| CIP1. 我计划在未来持续参加医疗保险，而不是中断或退出。 *I plan to continue participating in medical insurance in the future rather than interrupting or withdrawing.* | □1 □2 □3 □4 □5 □6 □7 |
| CIP2. 即使短期内没有明显的医疗需求，我也倾向于保持医疗保险的连续性。 *Even if I do not have immediate medical needs, I intend to maintain continuous medical insurance coverage.* | □1 □2 □3 □4 □5 □6 □7 |
| CIP3. 我对未来维持医疗保险所需的经济投入有较为清晰的认识。 *I have a clear understanding of the financial resources required to maintain medical insurance in the future.* | □1 □2 □3 □4 □5 □6 □7 |
| CIP4. 我已经对未来医疗保险相关支出进行了相应的财务规划或估算。 *I have made financial plans or estimates regarding future medical insurance-related expenses.* | □1 □2 □3 □4 □5 □6 □7 |

| **题项 / Item** | **作答 / Response** |
| --- | --- |
| AC2. 为了确认您正在认真阅读，请在本题选择“6—同意”。 *To confirm that you are reading carefully, please select “6 — Agree” for this item.* | □1 □2 □3 □4 □5 □6 □7 |

## **第4部分：基本信息 / Part 4: Demographic and Enrollment Information**

**D1. 您的性别是？** *What is your gender?*

□ 男 */ Male*	□ 女 */ Female*	□ 其他/自我描述 */ Other / self-describe*	□ 不愿透露 */ Prefer not to say*

**D2. 您的年龄是？** *What is your age?*

______________________________

**D3. 您的家庭户籍类型是？** *What is your household registration type (hukou)?*

□ 城镇户口 */ Urban*	□ 农村户口 */ Rural*	□ 其他/不确定 */ Other / Not sure*

**D4. 您的最高受教育程度是？** *What is your highest level of education?*

□ 初中及以下 */ Junior high school or below*

□ 高中/中专/职高 */ Senior high school / technical or vocational school*

□ 大专 */ Junior college / associate degree*

□ 本科 */ Bachelor’s degree*

□ 研究生及以上 */ Postgraduate degree or above*

**D5. 您目前的主要就业状态是？** *What is your current primary employment status?*

□ 企业职工 */ Enterprise employee*

□ 机关/事业单位或公共部门职工 */ Government / public-sector employee*

□ 个体经营/自主创业 */ Self-employed / business owner*

□ 灵活就业/零工 */ Flexible employment / gig worker*

□ 务农 */ Farming*

□ 学生 */ Student*

□ 无业/待业 */ Unemployed / not currently working*

□ 退休 */ Retired*

□ 其他 */ Other*

**D6. 您目前个人月收入大约是多少？** *What is your approximate current monthly personal income?*

□ 无固定收入 */ No regular income*

□ 少于2,000元 */ Less than RMB 2,000*

□ 2,000–3,999元 */ RMB 2,000–3,999*

□ 4,000–5,999元 */ RMB 4,000–5,999*

□ 6,000–7,999元 */ RMB 6,000–7,999*

□ 8,000–9,999元 */ RMB 8,000–9,999*

□ 10,000元及以上 */ RMB 10,000 or above*

## **结束语 / Closing**

**感谢您抽出时间完成本问卷！您的回答将帮助我们更好地理解影响长期医疗保障准备与持续参保判断的心理因素。再次感谢您的支持。 *Thank you for taking the time to complete this questionnaire. Your responses will help us better understand the psychological factors related to long-term medical protection and continued insurance participation. We sincerely appreciate your support.***

# **D. 问卷平台逻辑与变量命名建议 / Survey Platform Logic and Variable Naming**

以下内容仅供研究团队建卷与导出数据时使用。 *The following section is for survey programming and data export only.*

## **D1. Study 1 跳转逻辑 / Study 1 Routing**

- C1=不同意 → 结束；S1=否 或 S2=否 → 结束。 *C1 = decline → terminate; S1 = No or S2 = No → terminate.*
- A: IG=1, RD=1；B: IG=1, RD=-1；C: IG=-1, RD=1；D: IG=-1, RD=-1。 *A: IG=1, RD=1; B: IG=1, RD=-1; C: IG=-1, RD=1; D: IG=-1, RD=-1.*
- 情境后统一进入 FO1–FO4 → IG1–IG7 → RD1–RD5 → AC1 → D1–D6。 *After the scenario, route all participants to FO1–FO4 → IG1–IG7 → RD1–RD5 → AC1 → D1–D6.*

## **D2. Study 2 跳转逻辑 / Study 2 Routing**

- C1=不同意 → 结束；S1=否、S2=否、S3≠是 → 结束。 *C1 = decline → terminate; S1 = No, S2 = No, or S3 ≠ Yes → terminate.*
- 符合条件 → IG1–IG7 → FO1–FO4 → AC1 → RD1–RD5 → CIP1–CIP4 → AC2 → D1–D6。 *Eligible → IG1–IG7 → FO1–FO4 → AC1 → RD1–RD5 → CIP1–CIP4 → AC2 → D1–D6.*
- 建议导出隐藏变量：START_TIME、END_TIME、DURATION、CONDITION（Study 1）、SOURCE_CHANNEL。 *Recommended hidden export variables: START_TIME, END_TIME, DURATION, CONDITION (Study 1), SOURCE_CHANNEL.*

## **D3. 结束页文本 / Termination Page Text**

**感谢您的关注。根据本研究预先设定的纳入标准，本次无需继续填写。感谢您的理解与支持。 *Thank you for your interest. Based on the study’s pre-specified eligibility criteria, you do not need to continue with this questionnaire. We appreciate your time and understanding.***
